# Bilateral Idiopathic Dacryoadenitis in an Infant: A Case Report and Review of the Literature

**DOI:** 10.1155/crpe/5236578

**Published:** 2026-06-29

**Authors:** Jai Paris, Abdullah I. Almater, Khizar Rana, Theresa Power, James Slattery, Dinesh Selva

**Affiliations:** ^1^ Department of Ophthalmology & Visual Sciences, University of Adelaide, North Terrace, Adelaide, South Australia, 5000, Australia, adelaide.edu.au; ^2^ Department of Ophthalmology, College of Medicine, King Saud University, Riyadh, Saudi Arabia, ksu.edu.sa; ^3^ King Saud University Medical City, King Saud University, Riyadh, Saudi Arabia, ksu.edu.sa; ^4^ South Australian Institute of Ophthalmology, Royal Adelaide Hospital, Port Road, Adelaide, South Australia, 5000, Australia, rah.sa.gov.au; ^5^ Department of Anatomical Pathology, SA Pathology Women’s and Children’s Hospital, King William Road, North Adelaide, South Australia, 5006, Australia

**Keywords:** bilateral dacryoadenitis, idiopathic dacryoadenitis, lacrimal gland biopsy, noninfectious dacryoadenitis, paediatricorbital inflammation

## Abstract

Dacryoadenitis is most commonly caused by infectious, inflammatory or neoplastic processes. Idiopathic dacryoadenitis is exceptionally rare in children. We present a case of a 22‐month‐old previously healthy boy with progressive bilateral eyelid swelling, conjunctival chemosis and ocular discharge without systemic features. He was initially treated for atypical conjunctivitis but failed antimicrobial therapy and continued to deteriorate clinically. Investigations including bacterial swabs, viral serology and autoimmune testing were negative. Magnetic resonance imaging demonstrated bilateral lacrimal gland enlargement. Lacrimal gland biopsy revealed chronic lymphoplasmacytic inflammation without vasculitis or increased IgG4‐positive plasma cells. The patient was diagnosed with idiopathic dacryoadenitis and responded rapidly to systemic corticosteroids, with complete resolution within 3 weeks. This case highlights the diagnostic challenge of paediatric dacryoadenitis and the importance of early imaging and tissue diagnosis in atypical or progressive cases.

Dacryoadenitis, or inflammation of the lacrimal gland, is primarily infectious in paediatric populations [1]. While noninfectious aetiologies can be indicative of serious systemic conditions such as sarcoidosis, immunoglobulin G4‐related disease (IgG4‐RD) and granulomatosis with polyangiitis (GPA), they are typically associated with chronic dacryoadenitis and are rare in children [2, 3].

Children account for only 6%–17% of all orbital inflammatory cases, most commonly presenting with myositis, orbital pseudotumor or dacryoadenitis [4, 5]. Idiopathic dacryoadenitis, typically seen in middle age, is exceedingly rare in children. There are very few reported cases in the existing literature, all occurring in patients over the age of 6 years old [4]. We present a case of bilateral idiopathic dacryoadenitis in a 22‐month‐old male, without systemic involvement. To our knowledge, this is the youngest reported case of idiopathic dacryoadenitis. This case report was conducted in accordance with the principles of the Declaration of Helsinki. Written informed consent for publication of the clinical details and accompanying images was obtained from the patient’s parent/legal guardian. Institutional ethics approval was not required for this case report according to local requirements. A completed CARE checklist has been provided (Supporting Information (available here)).

## 1. Case Presentation

A previously healthy 22‐month‐old boy presented with a 4‐day history of progressive bilateral periorbital swelling, erythema, clear ocular discharge and irritation. Despite initial treatment for bilateral conjunctivitis with cefalexin (12.5 mg/kg) and chloramphenicol eye drops, symptoms failed to improve. There were no constitutional symptoms or prior medical history. The child had a SARS‐CoV‐2 infection 12 months prior and two negative tests since, including one just prior to admission.

Examination revealed bilateral periocular swelling, erythema, and clear discharge with significant palpebral and bulbar conjunctival chemosis (Figure 1A,B). There were no membranes or pseudomembranes and no corneal epithelial defects. Pupils were reactive to light. Extraocular movements were difficult to assess due to significant eyelid swelling. The child was afebrile and remained systemically well, with no lymphadenopathy. Atypical conjunctivitis was initially suspected, and azithromycin (10 mg/kg) was administered. Laboratory results showed an erythrocyte sedimentation rate of 15 mm/hr, C‐reactive protein of < 0.3 mg/L and white cell count of 9 × 10^9^/L.

**FIGURE 1:**
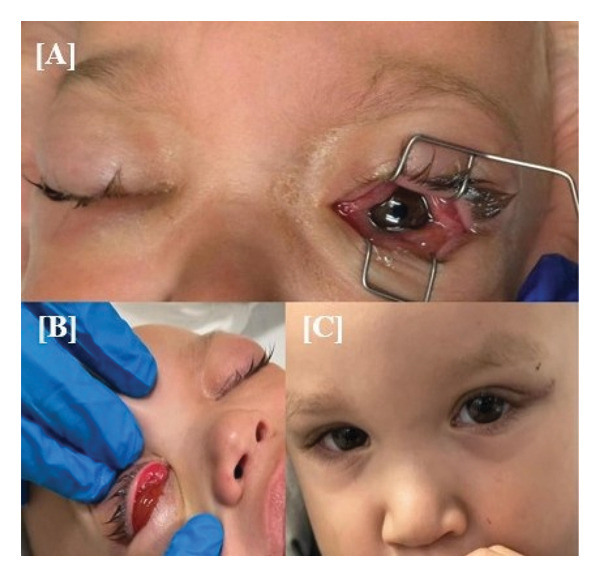
External photographs showing bilateral periorbital swelling, conjunctival chemosis, and crusting of clear discharge (A, B). Image C demonstrates significant resolution of periorbital swelling 3 weeks post presentation.

Despite 5 days of antibiotic therapy, there was continued clinical deterioration in chemosis and periorbital swelling. Orbital magnetic resonance imaging (MRI) was performed, which revealed bilateral pre‐ and postseptal soft tissue edema and lacrimal gland enlargement, suggesting dacryoadenitis with orbital inflammation (Figure 2). There was no abnormal scleral or optic nerve enhancement, and no abscess collection. Given the atypical bilateral presentation and progressive clinical course, a systematic evaluation for infectious, inflammatory and neoplastic causes of dacryoadenitis was undertaken. Bacterial swabs taken prior to the initiation of systemic antibiotics returned negative. Viral PCR, including adenovirus, enterovirus, HSV and VZV, also returned negative. Serology was negative for cytomegalovirus (IgM and IgG) and for Epstein–Barr virus (IgM 31.3 IU/mL (below cut‐off index) and viral capsid antigen IgG negative). Extensive autoimmune serological testing returned negative for rheumatoid factor, antinuclear antibodies, myeloperoxidase antibodies, proteinase 3 antibodies and angiotensin‐converting enzyme levels, with a weakly positive antineutrophil cytoplasmic antibody in a classical perinuclear pattern. IgG3 and IgG4 levels were mildly elevated (0.73 g/L and 0.292 g/L, respectively).

**FIGURE 2:**
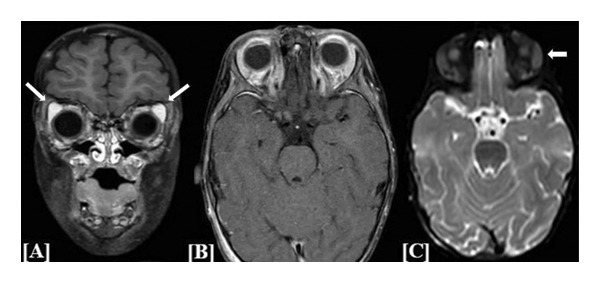
Coronal (A) and axial (B) T1‐weighted postcontrast showing bilateral lacrimal gland enlargement and enhancement (annotated). Axial diffusion‐weighted imaging (C) MRI showing increased diffusion in both lacrimal glands (annotated).

After three further days of clinical deterioration in periorbital swelling, a decision was made to proceed with diagnostic lacrimal gland biopsy (left) using an upper lid crease approach. This was undertaken to guide diagnosis and therapy, given the progressive clinical course, lack of response to antimicrobial therapy and unremarkable serological investigations. Histopathology revealed a patchy, predominantly lymphoplasmacytic, chronic inflammatory infiltrate with occasional eosinophils and scant intravascular neutrophils, as shown in Figure 3. There was no evidence of stromal fibrosis, granulomatous inflammation or vasculitis. Immunohistochemistry showed no increase in IgG4‐positive plasma cells. Flow cytometry showed no evidence of B cell monoclonality. The PCR analysis of the biopsy sample was negative for viral DNA. Following consultation with immunology, the mildly elevated IgG3/IgG4 levels were interpreted as a nonspecific finding in the context of systemic inflammation and absence of IgG4‐positive plasma cells on histopathology. Following consultation with rheumatology, the weakly positive ANCA in the context of negative PR3 and MPO without evidence of vasculitis on biopsy suggested a nonspecific finding and was not supportive of ANCA‐associated vasculitis.

**FIGURE 3:**
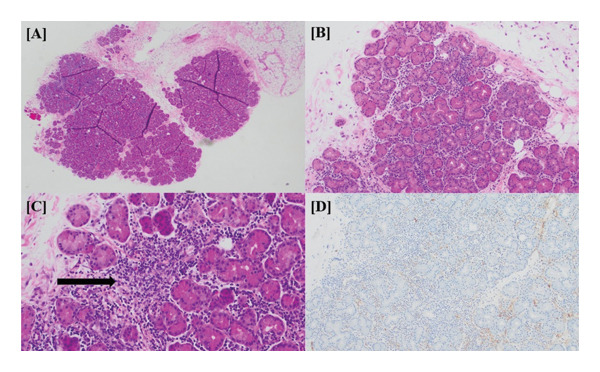
(A) Low‐power view (2×/0.08) showing normal lobular architecture of lacrimal gland tissue with preserved glandular elements and patchy chronic inflammation, with no significant fibrosis. (B) Intermediate‐power view (10×/0.4) highlighting a focus of inflammation. (C) High‐power view (20×/0.80) of the most affected area, demonstrating a predominantly lymphoplasmacytic infiltrate with scattered eosinophils; no evidence of phlebitis (annotated). (D) No increase in immunohistochemical staining for IgG (10×/0.4).

Given extensive microbiological, autoimmune and systemic inflammatory testing was unremarkable, a diagnosis of bilateral idiopathic dacryoadenitis was made. The patient was commenced on intravenous methylprednisolone (1 mg/kg daily for 3 days), followed by an oral prednisolone taper (12 mg for 2 weeks, 3 mg for 2 weeks and 1 mg for 2 weeks before cessation). There was rapid and complete resolution of symptoms within 3 weeks (Figure 1C), and the child remains well at 9 months post presentation. Both ANCA and IgG3/IgG4 levels normalized on repeat testing at follow‐up.

## 2. Discussion

Idiopathic dacryoadenitis is exceptionally rare in children, with few reported cases in the literature [4]. It remains a diagnosis of exclusion following systematic evaluation and is usually considered after clinical improvement to systemic steroid therapy. Noninfectious dacryoadenitis can occur as an ocular manifestation of systemic conditions, including sarcoidosis, GPA vasculitis, and IgG4‐RD, all of which have been documented in paediatric populations [6–8]. In paediatric populations, noninfectious dacryoadenitis mostly occurs in the disease spectrum of idiopathic orbital inflammation (IOI), although only 7% of paediatric IOI cases have clinical evidence of lacrimal involvement [5, 9, 10]. Our case is particularly notable for the exceptionally young age at presentation. A prior review of 68 paediatric idiopathic orbital inflammatory cases reported a minimum age of 2 years [11]. Smaller series focusing specifically on idiopathic dacryoadenitis demonstrate substantially higher median ages of onset compared to our case (11, 11.9 and 13 years) [4, 5, 11]. Typically, lacrimal gland enlargement in children is due to infective causes, which can be either bacterial or viral. Infectious dacryoadenitis has previously been reported in a 4‐month‐old infant with rhinovirus‐associated bilateral dacryoadenitis [12] and in a 10‐month‐old infant with presumed infectious dacryoadenitis responsive to antimicrobial therapy [13]. Noninfectious cases have been reported in a 19‐month‐old child with IgG4‐related dacryoadenitis [6] and an infant with IgG4‐related orbital inflammatory disease associated with IRAK‐4 deficiency [14]. In contrast, our patient demonstrated isolated bilateral dacryoadenitis with extensive negative infectious, autoimmune and histopathological investigations, supporting an idiopathic aetiology.

The clinical trajectory of idiopathic dacryoadenitis varies widely, with limited data on the nature of disease in young children. In adults, idiopathic dacryoadenitis can be acute or insidious and typically remains unilateral [15]. Disease severity ranges from isolated gland enlargement to extensive orbital inflammation involving extraocular muscles and fat, causing proptosis and motility issues [16, 17]. Ocular pain and extraocular movement restrictions are typical symptoms, but the absence of subjective complaints, as seen in our case, makes the diagnosis challenging in infants [11]. Idiopathic dacryoadenitis in children is unilateral in up to 87% of cases, making our case of bilateral gland involvement uncommon [5]. Approximately half of cases in the paediatric population are associated with constitutional symptoms (40%–50%); in contrast, our patient presented with no identifiable systemic symptoms [4, 5]. Belanger et al. reported systemic symptoms in 66.7% (4/6) of paediatric cases, including fever in 33.3% (2/6), upper respiratory symptoms in 16.7% (1/6) and weight loss and cough in 16.7% (1/6) [4]. Most cases also typically appear to involve multiple orbital structures, including the extraocular muscles, sclera, orbital fat or optic nerve [3–5, 11]. Isolated idiopathic lacrimal gland involvement, as seen in our case, is therefore exceptionally rare.

In children, dacryoadenitis can be challenging to diagnose clinically, often presenting with nonspecific features including eyelid swelling, ocular discharge or a superotemporal mass. Many cases may be misdiagnosed as infective conjunctivitis or orbital cellulitis, particularly if acute. A poor antibiotic response or atypical presentation should always prompt further investigation [2]. A structured diagnostic approach is essential, incorporating targeted microbiological testing, systemic evaluation for autoimmune or inflammatory disease and early imaging. Recommended serological evaluation includes inflammatory markers (ESR and CRP), autoimmune screening (ANA and ANCA), angiotensin‐converting enzyme levels, IgG subclasses including IgG4 and relevant viral serology (e.g., EBV and CMV). Orbital MRI is the preferred imaging modality, allowing assessment of disease extent and aiding differentiation of aetiology [18]. Infectious dacryoadenitis typically demonstrates unilateral diffuse gland enlargement with marked enhancement, surrounding fat stranding and possible extraocular muscle involvement [19]. Abscess formation, characterised by rim enhancement and restricted diffusion on DWI/ADC, strongly supports infection [20]. Some associated features can help support inflammatory causes, such as infraorbital nerve involvement or canal expansion which may suggest IgG4‐RD, or bony erosion, sinonasal involvement or optic nerve sheath thickening for ANCA‐associated vasculitis [19, 21]. Lymphoma often presents as homogeneous gland enlargement with low ADC values reflecting diffusion restriction [22]. In paediatric IOI, multistructure orbital involvement is common, often involving the superior rectus‐levator complex, orbital fat, lateral rectus, superior oblique tendon insertion and sclera [6, 12]. Lacrimal gland biopsy should always be considered to rule out infiltration from systemic diseases, provide a specific diagnosis, and guide targeted treatment prior to making a diagnosis of idiopathic dacryoadenitis. Histopathology also helps exclude infectious and malignant aetiologies, particularly when correlated with MRI [22], and is important for distinguishing lymphoid from sclerosing types, the latter indicating a worse prognosis [23].

IOI responds well to corticosteroid therapy, although the recurrence rates in children remain largely unknown. In adults with idiopathic dacryoadenitis, most demonstrate an excellent initial steroid response and disease recurrence occurs in approximately 13% of cases [24]. The treatment response in children is less well defined, though Belanger et al. report full resolution of symptoms in all of four cases treated with systemic corticosteroids. Long‐term monitoring for recurrence or transition to chronic low‐grade disease is recommended in all cases. There are reported cases of paediatric noninfectious dacryoadenitis failing initial therapy and requiring debulking orbitotomy [25]. Close inpatient monitoring during the acute disease phase is essential to ensure clinical regression following corticosteroid initiation.

This case highlights that idiopathic dacryoadenitis, although exceptionally rare in infants, should be considered in children with progressive bilateral eyelid swelling and poor response to antimicrobial therapy. Early orbital MRI and lacrimal gland biopsy are critical to exclude infectious, neoplastic and systemic inflammatory causes and guide timely corticosteroid treatment.

## Author Contributions

Jai Paris: conceptualisation, data collection, literature review, manuscript drafting, and revision of the final manuscript. Abdullah I. Almater: data interpretation, critical revision of the manuscript, and approval of the final version. Khizar Rana: clinical data acquisition, contribution to manuscript drafting, and critical revision. Theresa Power: histopathological analysis and interpretation, contribution to relevant manuscript sections, and critical revision. James Slattery: clinical supervision, contribution to study design and interpretation, and critical revision of the manuscript. Dinesh Selva: senior supervision, conceptual oversight, critical revision for intellectual content, and final approval of the manuscript.

## Funding

Open access publishing facilitated by Adelaide University, as part of the Wiley ‐ Adelaide University agreement via the Council of Australasian University Librarians.

## Disclosure

This work was previously presented in abstract form as a poster at the Royal Australian and New Zealand College of Ophthalmologists (RANZCO) Annual Scientific Congress 2025 and published as a conference abstract in *Clinical & Experimental Ophthalmology* (doi:10.1111/ceo.70045). All authors also attest that they meet the current ICMJE criteria for Authorship.

## Consent

Written informed consent for publication of the clinical details and accompanying images was obtained from the patient’s parent/legal guardian.

## Conflicts of Interest

The authors declare no conflicts of interest.

## Supporting Information

Additional supporting information can be found online in the Supporting Information section.

## Supporting information


**Supporting Information 1** Supporting Material 1 contains the CARE 2013 Checklist completed for this case report. The checklist has been provided to facilitate transparent and comprehensive reporting in accordance with CARE guidelines.
